# Perceptions of Cancer in Parents of Adolescent Daughters in Northwest Cameroon

**DOI:** 10.3390/curroncol30080519

**Published:** 2023-07-25

**Authors:** Laurie Elit, Eric M. Tum, Calvin Ngalla, Glenn M. Fungchwi, Joel Fokom Domgue, Elysee Nouvet

**Affiliations:** 1 Baptist Health Institute of Science, Mbingo P.O. Box 1, Cameroon; 2 Women’s Health Program, Cameroon Baptist Convention Health Services, Mbingo P.O. Box 1, Cameroon; ngalla.calvin@gmail.com; 3 Department of Obstetrics and Gynecology, McMaster University, Hamilton, ON L8V 5C2, Canada; 4 Information and Vocational Guidance Counselling Centre, Bamenda 5018, Cameroon; makiyighome@yahoo.com; 5 Pediatric Oncology, Cameroon Baptist Convention Health Services, Mbingo P.O. Box 1, Cameroon; mbahlos@gmail.com; 6 Department of Epidemiology, The University of Texas MD Anderson Cancer Centre, Houston, TX 77030, USA; jfokom@mdanderson.org; 7 Departments of Public Health and Obstetrics and Gynecology, Faculty of Medicine and Biomedical Sciences, University of Yaounde, Yaounde P.O. Box 812, Cameroon; 8 School of Health Studies, Western University, London, ON N6A 5C1, Canada; nouvete@gmail.com

**Keywords:** cancer, myth, perceptions

## Abstract

Background: Cancer is a rapidly rising cause of morbidity and mortality in sub-Saharan Africa. Cervical cancer, in particular, is still one of the leading causes of mortality for women in this setting. The uptake of healthcare services is in part influenced by patients’ belief systems. We sought to better understand the perception of cancer in the Kom tribe of Northwest Cameroon. Methods: A qualitative research study was completed using a semi-structured interview guide and one-on-one interviews with 45 parents of girls aged 9–14 years. These girls were candidates for free HPV vaccination to prevent cervical cancer. The interviews were recorded, transcribed, and analyzed using ATLAS.ti 9. Results: Thirty-five mothers and ten fathers with a median age of 42 yo were interviewed from Mbingo, Belo, Njinikom, and Fundong. Half of the parents were farmers, with three being herbalists or traditional medicine doctors. Seventy-seven percent had either no or only primary school education. None had had cancer. All knew at least one person with cancer. The most common word for cancer in the Kom language is “*ngoissu*”. It can refer to a bad infection or cancer. The occurrence of *ngoissu* is the result of either a curse placed on you, ancestral retribution, or transgressing the *ngoulatta* (snail shell spoken over and usually placed in a garden). The implications are that treatment of *ngoissu* must involve the traditional doctor who determines the spiritual issue and prescribes a remedy (like a herb or tea) and/or an incantation. Within the context of cancer, this can lead to a delay in diagnosis until the disease is no longer curable by conventional therapies. Conclusion: Ways to bridge biomedical healthcare services and traditional medicine are needed, especially in tribal contexts where the latter is an integral part of daily life.

## 1. Introduction

In Cameroon, cancer is a public health concern, with 15,000 new cancer cases annually and a prevalence of 25,000 cases [1]. The most common cancers in Cameroon are breast, cervical, non-Hodgkin lymphoma, prostate, and Kaposi sarcoma [1]. Humans rarely experience illness reduced to their biomedically significant features. As socio-cultural beings, we recognize, insert, and ascribe meaning to an illness within wide context-specific ways of understanding the world. As such, cancer, and indeed, any illness, can be said to exist in two realities: one involves the biological changes that are experienced in the human body. The other is related to how a person makes sense of such a diagnosis in accordance with its socio-cultural construction [2].

Culture refers to “a set of shared and socially transmitted ideas about the world that are passed down from generation to generation” [3]. As culture pertains to illness, “these beliefs and values influence perceptions around the meaning of an illness, the types of treatment that are useful and the likely outcome of health behaviors related to the prevention and control of disease” [3]. There are many ways cultural practices and beliefs impact health and disease in the population. Those of us from the West or Europe highly regard scientific medical knowledge and practice meaning as a coherent, universal, and objective account of health and disease. In contrast (especially in Africa), local and socially embedded practices of healing have a long history. Banwell points out that medicine is cultural, and this set of beliefs and behaviors impact disease prevalence and health outcomes. This has only been explored in a few specific ethnographic studies [4].

In addition to culture, religion or our “relationship of devotion or fear of God or gods” [5] results in a set of beliefs and behaviors that has an impact on disease. In other words, how people interpret events, resolve dilemmas, make decisions, and view themselves, their actions, and the actions of others influence behavior [6,7,8].

The healthcare system in Cameroon is structured predominantly through a government system of general and subspecialty hospitals with a minor component of care provided through private–public partnerships, private healthcare institutions, or religiously affiliated conglomerates. Healthcare is still procured overwhelmingly by a patient-pay-for-care system. For example, cervical cancer is the second leading cause of cancer death for women in Cameroon; however, cervical screening is not readily available or actively promoted, and treatment is only available at one private–public radiation facility that only has access to a Cobalt source. Vaccines are often freely available, and in 2021, free access to the HPV vaccine to prevent cervical cancer was only made available to 9-year-old girls. Vaccine uptake has only been 5%.

In 2015, the World Health Organization put forth the Sustainable Development Goals, and Goal 3 targets ensuring healthy lives and promoting well-being for all at all ages [9]. A component of this strategy would involve Universal Health Coverage to prevent catastrophic health expenditures. In Cameroon, this is no more evident than in those afflicted with cancer. Another component of SDG 3 involves healthcare being culturally relevant, in other words, considering local customs and integrating traditional health systems where appropriate [10].

In early 2022, our group had the opportunity to interview 45 parents of girls ages 9–14 yo concerning their knowledge, attitudes, and beliefs regarding the HPV vaccine. This vaccine had recently been made available free of charge by the government of Cameroon with the long-term intent to decrease the rates of cervical cancer and other lower anogenital tract and head and neck cancers. Our aim in the original work was to understand, from the perspective of parents, their reasons for or against vaccinating their pre-pubertal daughters against the cancer-causing Human Papillomavirus. In the process of building our interview guide, we undertook the exploration of the beliefs these parents had concerning the origin of cancer, their understanding of risk factors for cancer, and their beliefs and practices related to prevention and treatment. We did so based on the anthropologically informed premise that individual and community engagements with the HPV vaccine are contextual and depend on socio-cultural understandings of disease causality, treatment, and prevention. The study protocol and main findings on the HPV vaccine perceptions study have been published elsewhere [11,12,13]. The present article was developed to highlight findings in our data that cast light on Kom’s understanding of cancer.

To the best of our knowledge, there exists no previous research on Kom or Cameroonian understandings of cancer. This article adds to an extensive body of research asserting variability in meanings ascribed to cancer across cultures [14,15], with implications for public health decision-making, healthcare-seeking behaviors, and relationships between providers and the populations they serve. We anticipate findings presented here may be of use to healthcare providers, community leaders, or health promotion actors in Cameroon and will elaborate on implications for practice in the discussion.

## 2. Methods

### 2.1. Study Site and Population

This study [11,12] was conducted in the Mbingo region of Northwest Cameroon during a period of civil unrest. Participants were involved from a region located on the 24 km stretch of road from Mbingo to Fundong (Boyo Division), within the Boyo division. The approximate population is 75,000. The indigenous people group of this area are of the Kom kingdom; it is one of 250 indigenous tribes of Cameroon with a very much smaller component of Fulbe herders. These groups rely on subsistence farming and herding, respectively.

The Kom kingdom continues to be ruled by a Fon (king) even though there are parallel regional and national systems of government. Likewise, there is an embedded system of traditional healers and herbalists in this region [16], which we will refer to as indigenous medical practices. In parallel, there are biomedical facilities for diagnosis and healing, including 3 major hospitals in Fundong (Fundong Government Hospital), Njinikom (St Martin de Pores Catholic Hospital), and Mbingo (Mbingo Baptist Hospital), all with associated health clinics.

### 2.2. Study Design and Procedures

#### 2.2.1. One-on-One Interview

A detailed protocol for the HPV vaccine study has been published [11]. Qualitative data were obtained through one-on-one interviews with a medical anthropologist, and quantitative data from a short interviewer-delivered survey tool. The interviews were conducted from January to May 2022. Parents were recruited from the communities of interest through community healthcare workers. To be included, the individual had to be a parent of a daughter aged 9 to 14 years living in the Kom areas of Mbingo, Njinikom, and Fundong health areas. Individuals were excluded if they were a health worker or working in any health institution or if they were unable to converse in English or Pidgin English. Open inductive qualitative methods ensured that multiple perspectives were considered, and the quality of data gathered was maximized through triangulation. Written informed consent was obtained from each participant. We reinforced that their participation was optional and voluntary. Face-to-face in-depth interviews took place in a private office at the Mbingo hospital. A semi-structured interview guide was used [11]. Specific questions and probes were reviewed and refined during the research period in line with the themes that arose. Interviews lasted about an hour. All interviews were audio-recorded and transcribed in English.

#### 2.2.2. Data Analysis

Data gathering and analysis were a concurrent and iterative process [11,12]. The raw data were processed in their textual form and coded independently by 2 co-authors (CN, GF) to generate analytical categories of themes for further analysis with ATLAS.ti 9 (1993–2021 Scientific Software Development, GmbH Berlin, Germany) [17]. All the co-authors were involved in data analysis, and this was performed using the thematic analysis approach proposed by Braun and Clarke [18].

#### 2.2.3. Ethical Considerations

This study was conducted in line with ethical guidelines to protect the rights and welfare of all participants. All data were kept confidential and anonymous. Research approved was provided by the ethical committee of Hamilton Integrated Research Ethics Board, Hamilton, Canada (14022), and the Cameroon Baptist Convention Health Board Institutional Review Board (IRB2021-75). All participants were given detailed information about the objectives and methods of the research prior to their assent to be involved. They were given the opportunity to seek clarification at any time during the interview.

## 3. Results

The study population included 10 fathers and 35 mothers of vaccine-eligible girls; there was no relationship between interviewees (Table 1). The parents’ ages ranged from 27–72 years old (mean 41.9 yo) [12]. Mothers were younger (mean 39.5 yo) than the fathers (mean 50.4 yo). The prevailing tribal group represented was Kom (42/45). All participants spoke Pidgeon English or English. All parents identified as Christian except for one Fulani. No one identified as African tribal, but there appeared to be a syncretism of African tribal beliefs with Christianity. Predominantly, the parents only had a grade school education (76.7%). The most common job was farming (44.4%); other jobs included tailor/seamstress, marketplace vendor, or teacher. Within the group, in addition to their primary jobs, one identified as a traditional doctor and two as herbalists. None of the parents had had cancer, but 10/45 had known a close family member or friend with the disease.

### 3.1. What Is Cancer?

The scientific community recognizes cancer as an abnormal growth of cells with the potential to metastasize. Indeed, some of the participants described cancer as a wound that would not heal or a hard lump. The majority of participants in our study reverted to the Kom word *ngoissu* to describe cancer (27 parents). This word describes both a bad illness, such as infection, or cancer. Parents outlined several common characteristics of *ngoissu* as they used this term to describe what they had observed or heard about the condition. Many described *ngoissu* as causing a chronic internal headache (nine parents) associated with water or pus coming from a person’s ears (six parents). This problem was associated by parents with other abnormalities, including deafness (one parent), madness (one parent), neck stiffness (one parent), painful eyes (one parent), swollen stomach (one parent), or profoundly swollen legs (i.e., elephantiasis) (one parent). By convention, if the pus is coming from the ear, it is *ngoissu* (cancer) of the head; if pus is coming from the leg, it is *ngoissu* (cancer) of the leg. *Ngoissu* (cancer) is not linked to the cervix or prostate, as these organs are hidden.

Three parents elaborated that *ngoissu* can be wet or dry. Wet *ngoissu* is associated with a chronic boil or abscess (one parent). It can also be associated with a wound that does not heal (one parent) or vaginal discharge (one parent). Wet *nguossu* is most associated with stealing from a farm that is being protected by a snail shell that is filled with a concoction. Dry *ngoissu* is a hard nut or stone in the body. No boil is seen (one parent). Dry *ngoissu* occurs if someone steals from a farm that is being protected by an empty snail shell (one parent).

### 3.2. Transmissibility and Risks for Cancer

Seven parents believed cancer could be transmitted by coital activity (seven parents), via contact with a wound (two parents), mother-to-child, like HIV (two parents), inherited through genetics (three parents), or by sharing of bedding or towels (one parent).

Eleven parents did identify well-documented risk factors for cancer. These include smoking (nine parents), alcohol (six parents), herbicide and fertilizer effect on food (two parents), and/or burning toxic waste that pollutes the environment (one parent). Other risk factors that they listed were the use of marijuana (one parent), coins kept around the breast (one parent), maggi (salt-rich seasoning) (one parent), use of narcotic drugs (one parent), working too hard (one parent), too much stress (one parent), and heavy rains (one parent). Only one parent mentioned risk factors that are well known to be related to cervical cancer, including high gravidity, multiple sexual partners, and young age at first coitus. Other risk factors listed for cervical cancer were too much sex (one parent), lack of perineal cleanliness (one parent), and non-treated vaginal itch (one parent).

### 3.3. Warning Signs of Cancer

Eighteen parents commented on what they perceived as warning signs of cancer. In order of frequency, 12 noted that a lump or swelling in the breast, pus coming from any site (8), persistent headache (5), shortness of breath (1), weight loss (1), persistent pain (1), deafness (1), persistent noise in the ears (1), fever (1), change in skin colour (1) were signs of cancer.

### 3.4. Causes of Cancer

In addition to the scientific risks of cancer, the Kom people mentioned spiritual or mystical causes of cancer (two parents). The traditional doctor inflicts this disease by mixing a concoction (called *ngoullatta*) in the shell of a snail (called *ngoi*) (three parents). This is hung in an area like a farm (four parents). The goal of hanging the shell is to scare away thieves. If thievery happens, then the consequence is *ngoissu*. It is felt to be a very dangerous illness. Only traditional doctors can calm the illness.

While stealing from a farm was most associated (four parents) with *nguossu*, other transgressions could also be involved. These included adultery (one parent), rape (one parent), intentional urination with (two parents) or without menstruating (four parents) on a farm protected by the *ngoullata,* or wearing a red dress (four parents).

Other causes of cancer include ancestral retribution. This means that illness is from a curse or punishment on the living (15 parents) because the living abandoned the ancestors by not performing a traditional rite such as a death celebration or twin rite (*ekung*). Alternatively, the ancestors are not happy with an action of the living person as they have not respected social norms (four parents), like not respecting the will of a dying father or not continuing a family custom. Lastly, a third cause of cancer is when a curse is placed on a person (one parent).

This spiritual/psychological belief system is so strong that they believe modern medicine cannot remedy the spiritual aspect of the illness (one parent). The Kom rationalize that Africans treated themselves prior to the arrival of the white man (one parent), and so illness is within a spiritual context. Thus, a disease such as cancer is a dynamic process involving the person’s relationship to their social and cultural environment. A breach of moral precept results in the suffering of the responsible individual and cancer is sometimes attributed to guilt by a person, family, or village. In other words, illness results from displeasing the god(s) or the ancestor(s); an infraction of the moral law means a transgression against nature, and so the outlet nature is grief, and the consequence to the individual may be to develop cancer.

Some parents viewed illness as caused by both natural and supernatural factors at the same time. This may represent a blending of indigenous values and exposure to scientific practices, or it may just be two systems that are complimentary and not contradictory. For example, cancer because of a sexually transmitted disease (such as HIV) and a curse.

### 3.5. Treatment of Cancer

The Kom people group has indigenous beliefs, customs, and specialists associated with ensuring health and preventing and curing illness. Folk medicine practitioners like herbalists, tractional doctors, country doctors, bone-setters, diviners (*ngambe* man), and soothsayers are present in the community. Indigenous beliefs and practices can be secular, sacred, or both. Throughout this grass-field area of the northwest region, as is the case in much of sub-Saharan Africa, gathered plant parts are used to make teas, poultices, or powders to effect a cure. Self-treatment is often the first step in action.

Given this belief that cancer is caused by (1) transgressing against the *ngoullatta*, (2) ancestral retribution, or (3) from a curse placed on a person, the only cure for cancer is to consult the traditional doctor for they can discern the cause and what should be undertaken to remedy the situation (one parent). Sometimes the remedy is to drink a concoction. Other times there is a rite that must be performed on a stone. The stone is later used to grind away the cancer. If the rite is not performed, then anyone who crosses this stone will suffer from cancer (one parent). The physical act is accompanied by the performance of a ritual to prevent or heal an emerging illness episode [19]. The example given was that a child could not walk. The problem was that the family had not performed the dead celebration and enthroned a successor. Once this was carried out, the child was able to walk (one parent). The payment of spiritual debt is considered a way of treating cancer or other illnesses.

### 3.6. Prevention of Cancer

The Kom phrase for preventing an illness is *kente* (to defend) *kweh* (illness). Examples of the concept of prevention included scarification (three parents), charms or amulets (five parents), black thread tied to the arms of children to prevent meningitis during a meningitis outbreak (one parent), bathing in or ingesting herbs (three parents), or rubbing on leaves like pawpaw, guavo, or mango (two parents). The goal here is to protect the person spiritually from sickness. An example was that if a person with cancer is given a drink, then everyone in contact with the *ngoi)ssu* victim must drink the drink, or they will be afflicted with cancer (five parents).

Another method of prevention is by “*Sha’a*”, the Kom term for injection. This term was used by all respondents concerning vaccination. Other forms of prevention include fidelity, being faithful to the husband, abstaining from sex until married (one parent), and avoiding immoral acts like stealing (two parents). Here, one is adhering to a religious code, good morals, and correct cultural practices. This aligns with cancer as being a punishment for breaking a religious code or settling an intergenerational debt.

### 3.7. Role of the Traditional Doctor in Cancer Care

Traditional doctors are integrated into society. Some people see only a traditional or country doctor; others attend both a traditional doctor and hospital simultaneously; and some only access Western-type medical systems. Along the spectrum of prevention, diagnosis, and treatment, only one parent felt the traditional doctor has a role in **cancer diagnosis**. He indicated that based on the story of how the illness is manifesting itself, cancer can be diagnosed (one parent). In contrast, several parents felt that traditional doctors could not diagnose cancer, and so they were thieves.

Some parents felt that the traditional doctor could **treat** things that were not cancer (4/43 parents) or was cancer (7/43 parents) but could not cure cancer (16/43 parents). Some claimed not to know whether the traditional doctor has a role in managing cancer (4/43 parents). Some parents felt that it did not matter if you cured the cancer; the important factor was to treat the spiritual wrong (four parents). The sense was that at early stages, cancer could be cured (one parent), but at later stages, the goal of treatment was to alleviate pain (five parents) and calm the spread to other body parts (one parent). Apparently, not all traditional doctors can treat cancer. There are some that specialize in cancer treatment (for parents).

The agents that are used to treat cancer include vegetables (one parent); herbs (three parents); herbal teas like turmeric, black charcoal, and ginseng (two parents); leaves like black jack or *shweh* (three parents); grasses (one parent); or tree bark (one parent). The healing practice is combined with rituals, sacrifices, symbolic representations, or family or community support.

### 3.8. Role of Prayer in Cancer

Almost all (44/45) respondents identified as Christian, with one person being Muslim Fulbe. All respondents had an opinion concerning prayer in the context of cancer. There were 14/45 respondents who said prayer cannot heal a person, but combined with medicine, healing is possible (15 parents). Nine parents felt prayer could heal cancer. When cancer is related to evil spirits, two parents said that praying to God can chase those spirits away.

### 3.9. Role of the Hospital

Although local beliefs and cultural traditions coexist with biomedicine in this region, we see increasing use of healthcare facilities and increasing knowledge of modern medicine, especially by the younger generation and by those with higher education. The parents in our study articulated that the hospital’s role in cancer care involves providing information (4 parents), screening (1 parent), diagnosis (2 parents), and treatment (13 parents). Hospitals were an opportunity to access surgery (three parents) or medications like pain meds or chemotherapy (six parents). One parent said, “if you have cancer at an earlier state and is discovered at the hospital, it shall be treated. But if the cancer is chronic, it would be difficult for it to be treated at the hospital” (parent 10).

## 4. Discussion

In 2010, the people at Mbingo Cameroon said there had never been cancer until Dr. Richard Bardin (dually trained in Internal Medicine and Pathology) arrived [20]. This is in keeping with the newspaper report out of Ghana, which said that most of Africa’s 2000 languages have no word for “cancer” [21]. In that report, the writer alludes to cancer patients being stigmatized for doing something wrong and God punishing them. Consequent to this understanding, cancer management may be understood as requiring non-biomedical interventions, such as prayer, ritual, or incantations. In our study, we see the Kom people using a word used for cancer (*ngoissu*) that straddles potential diagnoses of infection or neoplasm. Probably the more important finding involves the implications of at least two paradigms. The indigenous paradigm pressures the family member to seek out the “*country doctor*” (traditional healer) to determine the reason for the bad thing happening and a way to fix the bad thing. The “*country doctor*” may be an herbalist who focuses on using teas or herbs. Alternatively, this can be a *ngambe* man who works through incantations and divination. Sometimes, the “country doctor’s” practice combines both therapeutic modalities. These individuals are a part of the fabric of the villages. Often the cost is not inconsequential and can involve payment for goods like a chicken. The other paradigm is a biomedical one, where the individual seeks out a diagnosis and subsequent treatment either through a local health center, regional government health facility, or faith-based hospital. In Cameroon, where all medical care is based on a patient-pay fee schedule, the cost of such care can be high. Our parents reported that prayer can often be a part of the medical management of cancer but not for the same purpose as in the first paradigm. The cause of cancer, the godhead which is sought, and the elements for disease diagnosis and management are clearly different.

The literature on the meaning of cancer from the perspective of an indigenous culture still in its original setting is sparse. A series of studies in the Muslim context highlights the role of the evil eye as a cause of cancer [22,23,24,25]. Two papers from Saudi Arabia showed that in a cohort of 952 patients from 2016 to 2018 [23], 33% mentioned the evil eye as the cause of cancer, up by 1.3% from the 2006–2008 cohort [22]. In this work, it appeared that the younger generation, those with male gender and those with jobs were more likely to discuss scientific causes of cancer.

Some of the literature on the meaning of cancer comes from survey data, so frequency information is available, but meaning or impact on behavior is lacking. Much of the literature on the meaning of cancer (usually with a specific type of cancer) comes from populations where the cancer patient has immigrated to a high-resource setting, and so it is not clear to what extent the beliefs and resulting behaviors have been influenced by their new context. The relevance of our work is understanding cancer and its impact on behavior by an indigenous culture that continues to exist in that indigenous setting. This understanding could inform strategies to move forward in improving healthcare, particularly cancer prevention and/or early detection in that setting.

### 4.1. Strengths and Limitations

The strengths of this work involve the use of the qualitative research design to explore questions about why or how. In this study, individual interviews were completed by a medical anthropologist from a neighboring community. He was facile with the customs and languages of the region. The rigor of design and analysis, including triangulation of methods, included member checking and meeting the STROBE criteria. Some limitations include those of qualitative methods, which include our inability to appreciate the degree to which these findings go beyond those interviewed and are pervasive in this region. In this study, we interviewed only those Kom who could speak English or Pidgeon English speakers, so we are missing perspectives of those speaking tribal languages, which may be more indigenous in their healthcare practices. Our main project objective involved the assessment of knowledge, attitudes, and beliefs concerning the HPV vaccine. This project gleaned this data as a secondary objective. The sampling process would have been different if this was the primary objective.

### 4.2. Clinical Implications

The implications of these findings are that families in an indigenous setting are pressured to seek out insight into the transgression once they experience symptoms. This pathway can lead to delays in accessing healthcare, which (as the key reason for delay amongst others, household/farm responsibilities, economics, and other crises) may result in the cancer being no longer curable [26,27,28]. The role of detecting precancer or an earlier stage of cancer does not appear to have a place in this paradigm. In the biomedical setting (without a spiritual element), several tests and referrals out of the community may be involved to make a diagnosis and obtain treatment. However, the cost is not inconsequential. Issues include direct and indirect patient costs, geography and implications of initial diagnosis and care away from the family unit, and not dealing with the spiritual element. Layered on this is the role of the government hospital in a region of civil war, whereas our respondents mentioned a lack of trust in the motives of the workers (i.e., death to the Anglophones (one parent)) and use of expired medicines (one parent). When gunshots are heard, the likelihood of travel toward healthcare is lower (one parent).

It remains to be explored whether economic hardship, distance from the healthcare facility, time of year (seasonal changes in access), and family responsibilities or gender impact whether hospital healthcare seeking is complemented with visits to traditional healers. Still, our findings do provide a first documentation and consideration of dominant, generally accepted indigenous beliefs about cancer amongst the Kom of Northwestern Cameroon. These beliefs are important to recognize from a public health perspective, as beliefs about the origins of disease can, especially if left unaddressed, limit effective prevention and management. Healthcare providers and public health promotion actors can ignore indigenous beliefs about cancer, but doing so risks shutting down opportunities for respectful dialogue and collaboration between such actors and the indigenous populations they are committed to serving. A more effective strategy, at least from this author group’s perspective, could be to respectfully acknowledge indigenous understandings of cancer, which includes understanding that cancer produces suffering, as an entry point to discussing what biomedical prevention and treatment measures offer.

Some health promotion and prevention strategies that would definitely improve access to biomedical care for the prevention and treatment of early-stage cancers include universal healthcare coverage of cost-effective strategies like HPV vaccination and cervical cancer screening. Educating the community healthcare workers on symptoms that should lead to immediate referral, like the project in Burkitt’s Lymphoma [29]. Educating the population about symptoms that should lead to healthcare. Improving the education of the population [22], especially women leads to their empowerment to seek effective prevention and treatment strategies. Partnering with religious communities, chaplains, and clergy for prayer but also meeting practical needs of families where a member is on the cancer journey.

## 5. Conclusions

We have highlighted the Kom tribe’s beliefs related to cancer and provided implications for disease management. Examples of respectfully bridging biomedical and traditional systems have been presented.

## Figures and Tables

**Table 1:** Demographics.

Characteristic	
Gender of the parents	
Female (mothers)	35 (81.5%)
Male (fathers)	10 (18.5%)
Age	
Mean (years)	41.9
50 years old and less	36 (80%)
51 and older	9 (20%)
Number of children (range)	1–34
Mean	5
Languages	
Kom	44
Pidgin English	38
French	3
English	2
Kedjon	1
Religion	
Christian	44
Muslim	1
Parental Education (Question answered by 43/45)	
None	1
Primary (Class 1–7)	
Class 3–5	5
Class 6/7	27
Secondary School	
Form 1–6	6
Post-Secondary school	2
Degree	2
Employment	
Farmer	20
Housewife	5
Tailor/seamstress	5
Teacher	4
Marketplace vendor/business	5
Other	6
Exposure to Cancer	
Personal history	0
Relative or close friend	10

## Data Availability

Trial registration: clinicaltrials.gov NCT05325138.
